# Adrenergic Signaling in Immunotherapy of Cancer: Friend or Foe?

**DOI:** 10.3390/cancers13030394

**Published:** 2021-01-21

**Authors:** Agnete Witness Praest Jensen, Ana Micaela Carnaz Simões, Per thor Straten, Gitte Holmen Olofsson

**Affiliations:** 1 National Center for Cancer Immune Therapy (DK-CCIT), Department of Oncology, University Hospital, DK-2730 Herlev, Denmark; agnete.witness.praest.jensen@regionh.dk (A.W.P.J.); ana.micaela.carnaz.simoes@regionh.dk (A.M.C.S.); 2 Department of Immunology and Microbiology, Faculty of Health and Medical Sciences, University of Copenhagen, DK-2200 Copenhagen, Denmark

**Keywords:** β-2 adrenergic receptor, β2AR, adrenergic receptor, exercise, physical activity, cancer, T cell, NK cell, β-blocker

## Abstract

**Simple Summary:**

Exercise is associated with many aspects of a healthy lifestyle. Among these, exercise leads to the secretion of adrenaline and noradrenaline, which mobilize cells of the immune system, a process which is suggested to possess therapeutic value in cancer therapy, alone or in combination with immunotherapy. Strikingly, administration of β-blockers—which block the effect of adrenaline/noradrenaline—are also suggested to be useful in cancer therapy alone or in combination with immunotherapy. Herein we discuss the question of whether exercise and the administration of β-blockers could potentially be useful in cancer therapy.

**Abstract:**

The incidence of cancer is increasing worldwide, which is to a large extent related to the population’s increasing lifespan. However, lifestyle changes in the Western world are causative as well. Exercise is intrinsically associated with what one could call a “healthy life”, and physical activity is associated with a lower risk of various types of cancer. Mouse models of exercise have shown therapeutic efficacy across numerous cancer models, at least in part due to the secretion of adrenaline, which mobilizes cells of the immune system, i.e., cytotoxic T and natural killer (NK) cells, through signaling of the β-2 adrenergic receptor (β2AR). Clinical trials aiming to investigate the clinical value of exercise are ongoing. Strikingly, however, the use of β-blockers—antagonists of the very same signaling pathway—also shows signs of clinical potential in cancer therapy. Cancer cells also express β-adrenergic receptors (βARs) and signaling of the receptor is oncogenic. Moreover, there are data to suggest that β2AR signaling in T cells renders the cell functionally suppressed. In this paper, we discuss these seemingly opposing mechanisms of cancer therapy—exercise, which leads to increased β2AR signaling, and β-blocker treatment, which antagonizes that same signaling—and suggest potential mechanisms and possibilities for their combination.

## 1. Introduction

The incidence of cancer is increasing worldwide, which is to a large extent related to the population’s increasing lifespan. However, lifestyle changes in the Western world are causative as well. To this end, smoking, obesity, excess energy intake based on processed food, red meat and fat, as well as physical inactivity, are on top of the list of risk factors (https://www.ncbi.nlm.nih.gov/books/NBK223925/). Considering these factors, smoking is more easily explained as a risk factor for lung cancer when it comes to mechanism of action, whereas most other lifestyle factors are more elusive in terms of their mechanism. Exercise is intrinsically associated with what one could call a “healthy life”. The World Health Organization (WHO) recommends that adults should exercise at least 150 min per week. The suggested exercise should consist of moderate-intensity aerobic physical activity in order to improve cardiorespiratory and muscular fitness, bone health and to reduce the risk of depression and noncommunicable diseases (NCDs) [1]. Nonetheless, 27.5% of all adults are physically inactive and thus do not meet the WHO global recommendation on physical activity and health [2]. The lack of physical activity has been suggested to increase the risk of NCDs including breast and colon cancers, coronary heart disease and type 2 diabetes by 6–10% [3]. Moore et al. pooled data from 12 prospective US and European cohorts with self-reported physical activity and concluded that physical exercise reduced the risk of ten cancers, including both solid and haematological cancers. The cancers in question were oesophageal adenocarcinoma; lung, kidney, colon, head and neck, rectal, bladder and breast cancer; myeloid leukaemia and myeloma [4]. The inverse associations for colon [5,6,7] and breast cancers [8,9] have previously been confirmed and are today acknowledge to be associated with physical inactivity. Interestingly, a study by Moore et al. showed that prostate cancer and melanoma were the only exceptions, for which exercise was demonstrated to have the opposite relationship. The latter was probably due to the fact that running or walking outdoors is frequently part of an exercise program and is inevitably associated with sun exposure [2]. Thus, exercise seems to confer protection against development of most, if not all, cancer types. Additionally, observational studies have shown that physically active patients with breast [10,11], colon [12], and prostate [13] cancer have statistically significant improved overall survival. In addition, numerous studies have firmly demonstrated effects in terms of quality of life (QoL), fitness and energy, as well as a positive impact on anxiety and depression [14]. Consequently, many oncology clinics include exercise as an embedded component in their standard of care to cancer patients.

In contrast to exercise, obesity is related to an increased cancer risk, most likely due to the fact that visceral fat secretes inflammatory mediators, e.g., IL-6 and CCL2 [15]. Strikingly, the increase in cancer incidence goes hand in hand with the current obesity epidemic [16]. However, the study by Moore et al. could demonstrate that the association between physical activity and increased cancer risk was BMI-independent [4]. Moreover, some data suggest that exercise can lower systemic inflammation, which points in the same direction [17]. This fits very well with the strong correlation between cancer risk and inflammation [18]. Exercise also leads to multiple changes in cardiovascular, metabolic and immune pathways. Pertaining to the latter, exercise has been shown to decrease levels of inflammatory cytokines in the elderly, and given the association between inflammation, obesity, sedentary lifestyle and cancer risk, it appears that exercise could be a key common denominator (https://www.ncbi.nlm.nih.gov/books/NBK223925/). Hence, understanding the mechanistic interplay between exercise, the immune system and cancer is of great interest.

As given in the above, there are strong data in support of a positive impact of exercise on cancer incidence, and also overall survival in cancer patients. In addition, data from mouse tumor models suggest that adrenaline, secreted in association with exercise, mobilizes immune cells, resulting in therapeutic efficacy. On the other hand, data are accumulating to suggest that β2-adrenergic receptor (β2AR) signaling in immune cells is detrimental to immune cell function, and along the same vein, that β2AR signaling in cancer cells supports intrinsic cancer cell traits. In the present review we focus on β-adrenergic receptors (βARs) and their ligands. It should be noted that other molecules released during exercise and/or stress, e.g., myokines and other hormones, have an impact on cells of the immune system as well as cancer cells. To this end, glucocorticoids are notable stress hormones with a complex biology, and a known impact on the immune system; knowledge and insight which is also exploited in cancer immunotherapy, as recently reviewed [19,20].

Nevertheless, in this review we summarize the apparently opposing β2AR data with an aim to help clarify if, when and why β2AR signaling is good or bad.

### 1.1. Cancer Immunotherapy and the Tumor Microenvironment (TME)

Cells of the immune system are capable of recognizing cancer cells, and therapies based on this capacity have been successfully exploited therapeutically. To this end, immunotherapies based on, e.g., administration of genetically engineered cancer specific effector cells (so-called CAR T cells [21]) or monoclonal antibodies (mAb) [22] that breach checkpoint inhibitory (CPI) molecules on immune cells, have revolutionized the treatment of disseminated cancers over the past decade. CPI has now been approved for treatment in numerous cancers, e.g., melanoma, head and neck cancer, non-small cell lung cancer (NSCLC), renal cell carcinoma (RCC) and bladder cancer, and in some diseases these immunotherapeutic drugs are now first-line treatments [23]. The fundamental mechanism of CPI therapy is unleashing the inhibition of spontaneously induced T and natural killer (NK) cell responses, allowing more efficient anti-tumor immune responses. An example is anti-PD-1/PD-L1 therapy, as PD-1 is typically expressed by activated T and NK cells and PD-L1 is typically expressed by cancer cells or cells of the innate immune system. Importantly, a fraction of patients on CPI therapy experience lasting complete responses, i.e., a cure from disease—this applies even to patients with disseminated late-stage disease [24].

Strong predictive markers to CPI therapy remain elusive. To this end, since PD-L1 is expressed by cancer cells, expression of PD-L1 on the target cells would make lot of sense to use as a predictive marker. Thus, the expression of PD-L1 has been scrutinized in numerous studies and is in fact used to select patients for treatment in some disease stages. For most cancers and disease stages, however, expression of PD-L1 is not a sufficiently strong predictive marker and most patients are treated irrespective of PD-L1 expression, or any other marker for that matter [25]. Other aspects of the tumor microenvironment (TME), pheno- or genotypes have been scrutinized in the search for predictive markers [25]. In colon cancer, the mutational burden has been shown to correlate with the response to therapy; only patients tested positive for microsatellite instability—indicative of high mutational load [26]—are treated with PD-1/PD-L1 CPI therapy. Supposedly, the high mutational burden renders cancer cells more immunogenic due to a relatively high fraction of neoantigens derived from gene mutations [25].

Cells of the immune system, i.e., T and NK cells, infiltrate tumors to various extents. Studies of these tumor-infiltrating lymphocytes (TILs), goes back more than 30 years, when Clark et al. defined these cells in melanoma and found that a “brisk” infiltration of T cells in primary lesions was associated with a favorable prognosis [27]. The term used nowadays, “hot tumor”, defines tumors comprising “many” CD8 T cells, whereas the term “cold tumor” defines tumors characterized by more limited numbers of CD8 T cells [28]. In recent years a major advance has been the development of the “immunoscore” to quantify CD3 (total T cells) and CD8 (cytotoxic T cells of defined phenotypes) T cells in a standardized and robust manner [29]. Additionally, the role of more protumor immune cell subtypes such as myeloid-derived suppressor cells (MDSCs) and regulatory T cells (Tregs) in the TME are also under investigation [30]. Importantly, the immunoscore has been shown to be a strong predictive tool of overall survival of patients with colorectal cancer, stronger than the conventional TNM system used for classification of malignant tumors [30]. The above data demonstrate that T cell infiltration into tumors—setting the stage for a “hot tumor”—may in fact be an important predictive marker for overall survival. Moreover, data are accumulating to suggests that patients with hot tumors are more likely to experience a response to CPI therapy [30]. Thus, finding ways to convert tumors from cold to hot has become a very important research topic, which could help increase response rates to CPI therapy and other forms of immunotherapies of cancer [31].

### 1.2. The Dual Effect of Adrenaline and Noradrenaline

We recently demonstrated in mouse cancer models that voluntary exercise, i.e., access to a running wheel, mediated the mobilization of immune cells and decreased the incidence and growth of tumors across several models. Strikingly, we could show that exercise led to an increase in numbers of immune cells infiltrating into tumors, and that blocking of the β2AR abolished the effect on cell mobilization, tumor influx of immune cells and therapeutic impact [32]. The main mechanism of action occurs via βARs (composed of three homologues subtypes; β1, β2 and β3) expressed by cells throughout the body, including cells of the immune system such as NK and T cells. βARs belong to a family of seven-transmembrane, G-coupled protein receptors, which are coupled to the G_s_ protein. In a canonical manner, activation of βARs leads to the activation of the G_s_ protein followed by cAMP synthesis. cAMP results in the activation of multiple downstream transcription factors through various intracellular signal transduction pathways, including protein kinase A (PKA) and guanine nucleotide exchange protein (EPAC) and thus extracellular signal-regulated kinase (ERK) 1 and 2, as well as the mitogen-activated protein kinase (MAPK) pathway [33]. The activated transcription factors subsequently result in cell modulation involving cellular and systemic metabolic pathways. 

**Figure 1:**
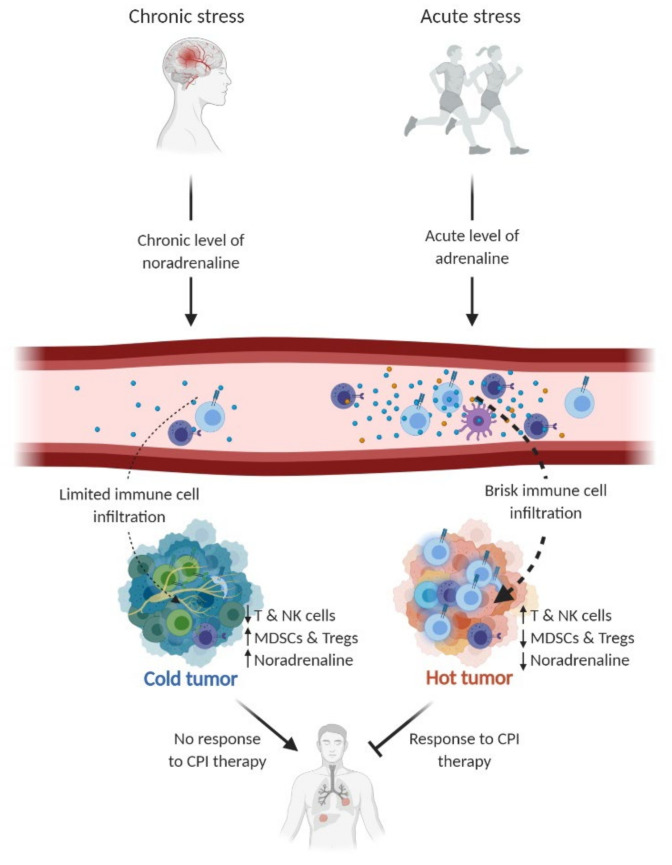
The dual effect of the catecholamines noradrenaline and adrenaline. Chronic stress leads to an increased baseline level of noradrenaline in the circulation and intratumorally, contributing to tumor progression and possibly suppression of anti-cancer immune responses. Conversely, acute stress (e.g., exercise) results in elevated adrenaline levels in the circulation, followed by instant mobilization and redistribution of immune cells. Preclinical studies have shown that exercise-induced mobilization increases tumor infiltration of immune cells, thus holding the potential of modulating the tumor microenvironment by converting it from a cold to a hot tumor. Consequently, this could increase anti-tumor immune responses and improve responses to checkpoint inhibitory (CPI) therapy. NK cells; natural killer cells. MDSCs; myeloid-derived suppressor cells. Tregs; regulatory T cells. Figure inspired by Hojman et al. [49].

During exercise, both ligands for these receptors—adrenaline and noradrenaline—are released mainly from the adrenal gland and sympathetic nerve terminals, respectively. Adrenaline and noradrenaline bind to the same βARs, however, with different affinity; adrenaline has a higher affinity to β2ARs, whereas noradrenaline has a higher affinity to β1ARs [34]. The binding to β2AR expressed by immune cells leads to mobilization of these cells to the blood stream [35]. After cessation of exercise, immune cells egress rapidly from the blood stream and immune cell frequencies may actually drop below baseline levels before reverting to normal in the span of a few hours [35]. This immunological part of the “fight-or-flight” response has supposedly evolved to allow more efficient immune responses as well as wound repair in response to damage suffered during a fight or flight [36]. Cancer cells also express βARs and thus agonists may influence cancer cell biology directly. To this end, it has been shown that β2AR signaling in cancer cells regulates a range of processes in cancer cells that contribute to the initiation and progression of cancer [33]. These data suggest that βAR signaling may in fact jeopardize anti-tumor immune responses and support tumor progression directly due to oncogenic signaling of β2AR. To this end, studies in mouse tumor models using antagonists of β2AR or taking advantage of β2AR knockout mouse models have both showed improved responses to CPI therapy [37]. Similarly, it has been shown that mice living under stressed conditions (too low housing) elicit weaker anti-tumor responses to tumors [37]. This could reflect more of a chronic stress condition, in which chronic levels of noradrenalin would be produced rather than acute levels of adrenalin, as seen in an exercise boost (Figure 1).

Additionally, there is also evidence to suggest that the βAR expression level is related to cell functional capacity. Following βAR activation, a desensitization process is initiated, resulting in reduced βAR cell surface expression and associated decreased responsiveness to further agonist stimulation [38,39,40]. The βAR desensitization only persists in the presence of βAR stimuli; hence, the cells recover in the absence of stimuli. The recovery time depends on the degree and duration of the receptor/agonist engagement [41]. This desensitization mechanism has been shown in healthy subjects who were treated with the β2AR selective agonist terbutaline, resulting in lymphocytes having a decreased βAR expression level [38,40]. Not only has this βAR downregulation been shown using agonist treatment, but also under psychological conditions. Yu et al. showed a correlation between lymphocyte βAR density and the subjects’ experience of anxiety and depression [42]. They further suggested that these changes in receptor expression affected the βAR responsiveness. To this end, Madden et al. showed in breast cancer cell lines that low βAR density resulted in decreased cAMP production and subsequently reduced downstream effects and vice versa [43]. Retrospective analysis on cancer patients undergoing β-blocker treatment (βAR antagonists) on its own [44,45] or in combination with immunotherapy [46] has suggested a clinical impact, but obviously prospective clinical trials are needed to support that notion. Promising data have emerged from early clinical trials [47,48]. Nonetheless, this strategy seems hard to comprehend in conjunction with the above data related to the positive effects of agonists, e.g., adrenaline, on anti-cancer immune responses, as shown in mouse tumor models [32].

### 1.3. βAR Signaling and the Impact on Cancer Cells

As mentioned, cells throughout the body express βAR for the catecholamines adrenaline and noradrenaline. Cancer cells both of hematological and epithelial origin express βARs, mainly the β2AR [50]. Downstream signaling of β2AR in cancer cells is associated with typical signaling traits of cancer cells [51], e.g., activation of pathways related to DNA repair, activation of oncogenes, angiogenesis, migration and inflammation [52].

As mentioned, exercise causes secretion of adrenaline and noradrenaline from the adrenal gland and sympathetic nerve ends. The same secretion pathways occur as a result of stress. The main source of noradrenaline in tumors is caused by sympathetic nerve activity, since many types of tumor tissues are innervated by sympathetic nerve fibers [53]. Thus, the level of noradrenaline is often higher in the tumor compared to serum, underscoring the local secretion [47,54]. To this end, it was recently demonstrated that circulating adrenaline was not required for chronic stress to enhance metastasis. Taking advantage of human xenografts as well as an immune-competent breast cancer mouse model, it was shown that the effects of chronic stress on cancer progression was independent of systemic levels of adrenaline [55]. In addition, Bucsek et al. demonstrated that chronic stress induced by lowering housing temperature from thermoneutral ambient temperature resulted in an elevated intratumoral baseline level of noradrenaline, followed by reduced intratumoral CD8 T cell frequency and functionality. This mechanism was blunted when βAR activation was prevented using either a non-selective β-blocker propranolol or β2AR knockout mice (Adrb2-/-) [56]. Kokolus et al. also showed that experimental mice kept at a thermoneutral temperature were associated with a reduction in tumor formation, growth and metastasis, and tumors were characterized by more pronounced infiltration of with CD8 T cells [57]. A recent paper by Perego et al. demonstrated an interesting connection between noradrenaline stimuli of polymorphonuclear neutrophils (PMNs)/MDSCs, resulting in reactivation of dormant tumor cells. The data suggest a mechanistic connection between noradrenaline, causing MDSCs or neutrophils to release the protein S100A8/A9, eventually causing tumor cells to exit their dormancy and form new tumor lesions. The effect was blunted by β-blockers. This is interesting because it offers a potential mechanistic description of noradrenaline’s effect on MDSCs and neutrophils in the TME [58]. These data support the notion that sympathetic nerve terminals represent a key provider of noradrenaline in the TME (Figure 1).

Given the widespread use of β-blockers, e.g., for treatment of arrhythmia, high blood pressure and anxiety, numerous retrospective studies have been conducted. Some studies could not demonstrate any effect, but the bulk of studies showed an association between the use of β-blockers and clinically meaningful effects, e.g., in terms of overall or progression free survival [56]. On example is Kokolus et al., who retrospectively studied 195 patients, showing that patients using β-blockers had prolonged survival upon immunotherapy [59]. In part due to the retrospective nature of these findings the mechanism of action is uncertain. It could be speculated that more information could be gathered if retrospective clinical data were combined, e.g., with studies of nerve innervation [60], expression of neurotrophic factors and βARs in the tumor, which could possibly be feasible based on archival material [61]. Interestingly, β2AR has been shown to shift between active and inactive conformations even in the absence of agonists, suggesting a level of background signaling [62]. Supposedly, this could play a role and maybe even more in cells that express high levels of the receptor, e.g., cancer cells.

Prospective studies are still quite few and small; hence, bigger studies are needed to be able to demonstrate clinical efficacy. To this end, De Giorgi conducted a two-armed prospective cohort study including 53 patients with melanoma, in which patients in the treatment arm (*n* = 19) received standard of care plus 80 mg propranolol daily. In this small cohort, data collected three years out demonstrated significant benefits in terms of progression free survival (PFS) in the propranolol arm [48]. More recently, Gandhi et al. treated nine melanoma patients with an increasing dose of propranolol, together with CPI therapy (pembrolizumab), and observed a response rate of 78%. Although being a very small trial—a phase I study, without a control group—these data are again encouraging [47]. Testing melanoma patients makes a lot of sense, because pre-clinical data from mouse melanoma models as well as retrospective data [59] suggest efficacy, also in combination with immunotherapy. Moreover, melanoma cells express very high levels of β2AR [50].

Data from mouse tumor models have shown that stress can accelerate tumor progression in a range of cancer models [51]. In fully immune competent models, the involvement of the immune system cannot be excluded, but some studies have used immune compromised mouse models, e.g., nude or NSG/SCID mice, to establish that β2AR signaling contributes to tumor progression independently of T cells [63,64] and T, B and NK cells [65,66]. Supporting the notion of the direct involvement of stress-associated levels of β-agonists in tumor progression, administration of the same agonists in tumor mouse models have been also shown to promote tumor progression [52], and to compromise the effect of chemotherapy [67,68].

Summing up, β2AR signaling in cancer cells seems to contribute to cancer progression and retrospective data suggests that the use of β-blockers may improve clinical outcomes in cancer in terms of overall survival. Very few data are yet available from prospective clinical trials but data from small phase I trials are encouraging.

### 1.4. βAR Signaling and the Immune System

As stated above, clinical trials are underway to test the notion of administering β-blockers to cancer patients alone or in combination. Cells of the immune system also express the β2AR most pronouncedly in NK cells, but also in T cells and cells of myeloid origin. The high expression levels of β2AR by NK and T cells are reflected in the fact that these cells are mobilized most dramatically upon acute increases in adrenaline levels, e.g., during exercise [69,70]. During exercise, contracting skeletal muscles secrete myokines, many of which are cytokines with key functions in the immune system. One example is IL-6, which is secreted by muscles during exercise, and it was recently shown that IL-6 receptor blockade in exercising volunteers by administration of tocilizumab led to a significant decrease in the mobilization of NK cells and dendritic cells. This strongly suggests that muscle-derived IL-6 plays an important role in exercise-induced mobilization of immune cells [71]. Preferentially mobilized leukocytes are central memory, effector memory and terminally differentiated CD8 T cells and CD56^dim^KIR^+^/NKG2A^−^ NK cells [69]. Mobilization of these lymphocytes leads to redistribution within different body compartments [72], which has been shown to enhance the immune function in the skin [73]. Whether it occurs at all sites to which immune cells traffic during acute stress is debatable. Exercise can therefore potentially support/replace exhausted lymphocyte cells in peripheral tissues with activated lymphocytes which are a “better fit”.

When it comes to the functionality of mobilized cells, multiple in vivo and in vitro studies have shown opposing effects of adrenaline signaling in lymphocytes. Thus, some studies have demonstrated that adrenaline signaling has a positive effect on lymphocytic cells. In this regard, regular exercise has been shown to reduce the risk of infection and the burden of latent viral infections [74]. Exercise has also been shown in clinical trials to improve vaccination-induced immune responses to both novel and known antigens [75]. However, some data suggest that exercise increases the risk of infection, maybe reflecting differences in the intensity and duration of exercise, as well as the readouts used to evaluate immune capacity. However, there is some consensus that short/moderate intensity for up to 45 min is beneficial for host immune responses [76].

At the cellular level—with a prime focus on T and NK cells—several in vitro studies have been conducted to scrutinize the effects of exercise. To this end, LaVoy et al. demonstrated that mobilization of T and NK cells by exercise was intensity-dependent and that mobilized T and NK cells secreted increased amounts of cytokines (IFN-γ, IL-2, IL-4 and IL-10) when analyzed ex vivo [70]. Similarly, naïve murine CD4 T cells were demonstrated to produce two to four times more IFN-γ per cell upon reactivation in the presence of noradrenaline [77]. Interestingly, when βAR agonist was added prior to or during T cell activation, the IFN-γ secretion was decreased, whereas when it was added after activation, IFN-γ production was increased. These data indicate that the time point of βAR signaling influences lymphocyte activation and thus cytokine secretion.

As exemplified above, some studies suggest that exercise adds to the functional capacity of immune cells tested ex vivo or studied upon addition of β2AR ligands. However, the bulk of studies have also shown that exercise—and adrenaline signaling—hampers the functional capacity of the cell. Earlier studies have examined the link between IL-2 production and β2AR expression in CD8 T cells. IL-2-stimulated CD8 T cells were shown to increase their β2AR expression level, rendering them more sensitive to β2AR stimulation [39]. Simultaneously, β2AR activation was shown to suppress the production of IL-2, expression of the IL-2 receptor (CD25) and subsequently proliferation [78]. A more recent study performed with isolated human CD8 T cells also showed a reduction in IFN-γ and TNF-α secretion upon activation in the presence of noradrenaline or the β2AR agonist salmeterol [79,80]. In addition to cytokine production and proliferation, Qiao and colleagues have shown that β2AR activation also impairs the normal activation of murine CD8 T cells in vitro by suppressing required metabolic reprogramming events. This resulted in downregulated glucose transporter 1 (GLUT1) expression, decreased glucose uptake and glycolysis, and finally impaired mitochondrial function [81].

As exemplified above, a wide body of literature has investigated the effect of βAR agonist on T cells through in vitro analysis. More limited in vitro studies have been conducted to scrutinize how βAR signaling affects NK cell functionality, but a similar suppressive effect on NK cells as for T cells has been suggested. Thus, Wallace et al. showed that NK cells incubated with salmeterol and subsequently activated by the MHC-I-deficient erythroleukemic cell line K562 had reduced CD107a expression, suggesting that NK cell cytotoxicity is suppressed by β2AR stimulation [80]. Importantly, this was only significant at a very high concentration of salmeterol (50 µM). Another study examined TNF-α and IFN-γ secretion in activated human NK cells, which was also shown to be reduced [82]. Sarkar et al. further indicated reduced NK cell cytolytic activity of splenic lymphocytes due to downregulated perforin, granzyme B and IFN-γ [83]. In contrast, Strannegård and colleagues demonstrated that βAR signaling has a dual influence on human NK cell activity. They showed that NK cells pre-treated with a low βAR agonist concentration prior to K562 NK cell lysis assay increased NK cell activity. On the other hand, the addition of a high concentration of βAR agonist during NK cell activation inhibited their activity [84]. Thus, although data are more scarce concerning the functional impact of β2AR signaling, the available data suggest a suppressive role of β2AR agonists.

Overall, the data point towards βAR signaling having a negative impact on T and NK cell functionality. It is, however, important to address the relative simplicity of in vitro studies in relation to the biological complexity. For instance, most of the in vitro studies used unphysiologically high levels of βAR agonist (1 µM–1 mM) compared to adrenaline and noradrenaline concentrations released during exercise (<2500 pg/mL adrenaline and <8000 pg/mL noradrenaline (unpublished data)). Other influential factors are the non-specific stimulation of the cells and the exclusion of a natural milieu. To this end, as noted previously, exercise leads to a marked increase in numerous cytokines and myokines, e.g., IL-6, which was shown to have a huge effect on cell mobilization [71]. These methodological obstacles may lead to inconsistencies between in vitro and in vivo studies and the biological understanding of how adrenaline and noradrenaline impact immune cells.

### 1.5. Mechanism of AR Signaling in the Context of Exercise Oncology 

Although other species have been used, the most frequently used animal in studies of exercise, as well as oncology and immunology, are mice. A variety of tumor models in mice—i.e., chemically induced, transplantable (subcutaneous or orthotopic) or spontaneous/genetic—have been used to scrutinize the impact of exercise on tumor formation, progression and outcome. Two excellent reviews recently summarized the available data in this field [85,86], concluding that most studies could demonstrate an anti-tumor effect of exercise, however, some studies showed no or even the opposite effect. We will not go into further detail here since these issues are well discussed in the mentioned reviews [85,86]. It does, however, highlight that literature in this field should be evaluated with caution. Moreover, studies that are merely descriptive and inadequate in terms of mechanistic insight seem less advantageous to the field in terms of translational value as well as drug development.

We studied the effect of voluntary exercise (i.e., access to a running wheel) in the C57Black mouse strain and demonstrated a decrease in tumor incidence of a chemically induced liver cancer model, as well as in the spontaneous melanoma mouse GRM-1. A significant effect was also found in transplantable B16 melanoma and Lewis lung cancer for both upon subcutaneous inoculation and for B16 also lung “metastases” after tail vein injection. Concerning the mechanism of action, we studied subcutaneous B16 tumors in more detail, and showed that exercise led to the release of adrenaline, which led to the mobilization of immune cells, most notably NK and T cells, and increased the influx of immune cells in the tumor [32]. Blocking of β2AR using propranolol abolished the effect completely, as did clearing of NK cells from the animals. Importantly, the effect could in part be mimicked by i.p. injections of adrenaline [32]. Thus, in this model β-blockers in fact blocked the therapeutic response.

Other recent studies have demonstrated the therapeutic effect of voluntary exercise using running wheels. Thus, endurance training in running wheels slowed down the progression of 4T1 breast cancer tumors, corresponding with a decrease in numbers of FoxP3+ Treg cells at the tumor site. In addition, the crucial involvement of T cells was substantiated by the absence of effects in athymic mice [87]. Wennerberg et al. similarly demonstrated the therapeutic effect of exercise using mice exercising in running wheels. Tumors from exercising mice have an immune cell infiltrate with a lower frequency of MDSCs and a more favorable CD8/CD4 ratio [88]. Treg cells were not studied, but the change in CD8/CD4 ratio could potentially be in part due to fewer Treg cells.

A recent study scrutinized the efficacy of OT-1 specific T cells harvested from exercising mice (access to running wheels). Strikingly, these T cells showed a change in metabolism, and were more effective in adoptive transfer studies. Surprisingly, the effect of exercise could be mimicked by daily infusions of lactate [89]. Although the latter finding is unique, all the abovementioned studies showed that exercise mobilized cells to the tumor mass, i.e., converted the cold tumor to a hot tumor.

### 1.6. β-Blockers and Exercise in Cancer Treatment

We and others are currently testing in clinical trials if exercise could be a tool to increase the influx of immune cells in patients, improving the chance of a response to immunotherapy (www.clinicaltrial.gov). In this context, we also aim to improve the understanding of the underlying mechanisms of these potential effects. In August 2020, we initiated a randomized controlled trial (RCT) called “High-Intensity Aerobic exercise training and Immune cell Mobilization in patients with lung cancer (HI AIM)” (NCT04263467). In short, the overall purpose of this study is to investigate if medium- to high-intensity training can mobilize cells of the immune system, in particular T and NK cells, and thereby potentially enhance the infiltration of lymphocytes into the tumor, enabling a response to PD-1 CPI therapy. The HI AIM trial will include 70 patients with metastatic NSCLC. Patients in the intervention group will participate in a six-week exercise intervention, consisting of medium- to high-intensity exercise training sessions three times per week. Each training session will primarily focus on interval training using a bike ergometer. All exercise sessions will be conducted in groups and supervised by a nurse and physiotherapist. Patients participating in the study will receive concurrent standard treatment, i.e., CPIs, CPIs combined with chemotherapy or oncological surveillance.

As discussed in this review, some of the key focuses in terms of understanding the interplay between the immune system and exercise oncology are adrenalin and noradrenaline’s roles in immune cell mobilization and the TME. We aim to focus on these key questions when monitoring the effect of the HI AIM trial. For this reason, blood samples will be obtained pre- and post-exercise and over the course of the trial, and biopsies will be performed before and after the exercise intervention when available. For the first part, we have conducted several optimization steps for blood sampling (inspired by the publication by Rooney et al. [35]), showing a fast egress of lymphocytes and monocytes (within a few minutes) after the end of exercise. These data underscore the importance of drawing blood samples immediately after the cessation of exercise. Data from our lab confirm the high degree of mobilization of immune cells at two minutes with a rapid decline already at five minutes and ten minutes (unpublished data). Hence, all our blood samples taken post-exercise will be taken within two minutes after the completion of exercise.

As mentioned, after high-intensity training the frequency of peripheral T cells, NK cells, γδ T cells, monocytes and granulocytes are known to increase. For patients with NSCLC, a high neutrophil-to-lymphocyte ratio [90] and a high frequency of MDSCs [91,92,93] have been associated with reduced overall survival and response to treatment, respectively. Therefore, to investigate the mobilization of neutrophils and MDSCs into the peripheral blood of NSCLC patients after exercise, we designed a multicolor flow cytometry panel focusing on different immune populations. Thus, one of our flow panels was aimed at characterizing the different populations within granulocytes (neutrophils, eosinophils, basophils and mast cells) and MDSCs (monocytic-MDSC (mMDSC), granulocytic-MDSC (gMDSC) and early-MDSC (eMDSC)). The evaluation of granulocytic populations will be conducted on whole blood, as previously reported for patients with NSCLC [94]. 

To determine reliable measurements of adrenaline and noradrenaline levels in the blood, this method was also tested and optimized. Testing several parameters such as serum vs. plasma, handling time and handling temperature of samples, our data showed that adrenaline and noradrenaline should be measured in serum samples, handled at room temperature and most importantly, samples should be frozen within one hour after blood collection (unpublished data).

Several lines of evidence have suggested that the use of β-blockers lowers the risk of cancer. Moreover, as discussed above, cancer cells express βAR and downstream signaling is associated with typical cancer traits. β-blockers have different affinity to βARs and hence are often classified as either selective (antagonists with a great affinity towards β1ARs) or non-selective (antagonists which bind β1- and β2ARs with equal affinity) [95]. Regardless of their known selectivity, little is known about the effectiveness in cancer treatment of the individual compounds. Today, most of our knowledge of β-blockers in cancer treatment is based on retrospective epidemiological studies, which have not taken the individual compounds’ properties into account. To this end, β-blockers have recently gained interest in the field of oncology, leaving us with no comparative clinical trials of β-blockers in cancer treatment and hence it is difficult to make specific recommendations. To the best of our knowledge, only one retrospective study has compared the use of selective and non-selective β-blockers in patients with epithelial ovarian cancer (*n* = 269). They concluded that patients using non-selective β-blockers were associated with longer overall survival [96]. This discovery supports our understanding of β2AR signaling on cancer progression. Graff et al. showed under controlled conditions that healthy volunteers preferentially mobilized matured CD8 T and NK cells via β2AR activation during a bout of exercise [69]. This was shown by the use of a β1-selective antagonist, bisoprolol, and a non-selective antagonist, nadolol. These data suggest that β2AR signaling has a central role for both β-blocker and exercise cancer treatments.

**Figure 2:**
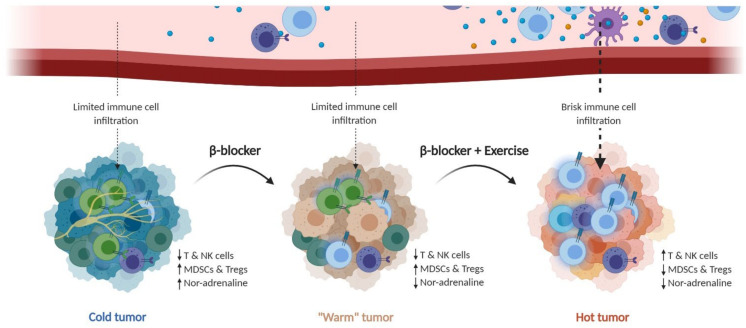
β-blockers and exercise: can they be combined/work together? Adrenergic signaling is a double-targetable checkpoint in immunotherapy of cancer. Adrenergic signaling seems to be dependent on multiple factors, such as acute and chronic stimuli. Based on mice studies, there is evidence to suggest a therapeutic benefit from both blocking chronic adrenergic signaling by β-blockers, as well as using the advantages of acute adrenergic signaling by exercise. We therefore hypothesize that adrenergic signaling can be a double-targetable checkpoint pathway, with one target being chronic noradrenaline stimulation of β-adrenergic receptors in the tumor microenvironment by administering β-blockers, which may turn a cold tumor to a “warm” tumor. The other target utilizes the positive effect of acute adrenergic signaling by exercise, leading to exercise-induced mobilization and tumor infiltration of cytotoxic lymphocytes (T and NK cells), thus turning the “warm” tumor into a hot tumor. Simultaneously β-blockers and exercise could be used as combinatorial “sequence” treatments, together with the respective immunotherapy cancer treatment. NK cells; natural killer cells. MDSCs; myeloid-derived suppressor cells. Tregs; regulatory T cells.

The main therapeutic mechanism of exercise, as well as the use of β-blockers, seems to occur via effector cells of the immune system. Thus, it appears that blocking as well as pushing signaling through β2AR in immune cells can be therapeutic through different mechanisms. β-blockers appear to unleash T cells from suppressive β2AR signaling, installed by chronic high levels of noradrenaline in the TME, whereas exercise mobilizes immune cells by increased acute levels of adrenaline, which in turn leads to increased immune cell infiltration in the tumor. Although more needs to be learned about the mechanisms involved—be it in β-blockade or exercise—it could open combination possibilities in which the use of β-blockers is interrupted by short periods—days—of intense exercise (Figure 2).

With clinical trials ongoing—alongside continued studies in mouse tumor models—the near future will bring exciting new insights onto the role of adrenaline/noradrenaline, exercise and β2AR signaling in cancer. Additionally, this will provide knowledge about how it influences immune cell subsets and the TME, and importantly, how it impacts on the lives of patients.

## 2. Conclusions

Adrenergic signaling in the immunotherapy of cancer: is there a perfect regimen that exploits the mobilizing capacity of adrenergic signaling upon acute exercise, while at the same time allowing the blockage of the damaging chronic adrenergic signaling in the TME? Potentially this could be achieved if β-blockers and exercise were combined in sequence, thereby blocking the chronic adrenergic signaling in the TME—interspersed with mobilization events leading to the influx of immune cells into the tumor. This strategy could hold the potential to improve the mobilization of immune cells to the tumor, while also unleashing T and NK cells from the suppressive action of chronic adrenergic signaling in the TME. Still, further data are needed, experimentally and clinically, and it will be interesting to follow future studies clarifying the mechanistic roles of how both exercise and β-blockers influence cancer and the immune system.
